# Prostate cancer diagnosis based on multi-parametric MRI, clinical and pathological factors using deep learning

**DOI:** 10.1038/s41598-024-65354-0

**Published:** 2024-06-28

**Authors:** Haniye Sherafatmandjoo, Ali A. Safaei, Foad Ghaderi, Farzad Allameh

**Affiliations:** 1 Department of Data Science, Faculty of Interdisciplinary Science and Technology, Tarbiat Modares University, Tehran, Iran; 2 Department of Medical Informatics, Faculty of Medical Sciences, Tarbiat Modares University, Tehran, Iran; 3 Human‑Computer Interaction Lab, Electrical and Computer Engineering Department, Tarbiat Modares University, Tehran, Iran; 4 Laser Application in Medical Sciences Research Center, Shahid Beheshti University of Medical Sciences, Tehran, Iran

**Keywords:** Prostate cancer, Deep learning, Magnetic resonance images, Convolutional neural networks, Clinical and pathological data, Diagnosis, Medical imaging

## Abstract

Prostate cancer is one of the most common and fatal diseases among men, and its early diagnosis can have a significant impact on the treatment process and prevent mortality. Since it does not have apparent clinical symptoms in the early stages, it is difficult to diagnose. In addition, the disagreement of experts in the analysis of magnetic resonance images is also a significant challenge. In recent years, various research has shown that deep learning, especially convolutional neural networks, has appeared successfully in machine vision (especially in medical image analysis). In this research, a deep learning approach was used on multi-parameter magnetic resonance images, and the synergistic effect of clinical and pathological data on the accuracy of the model was investigated. The data were collected from Trita Hospital in Tehran, which included 343 patients (data augmentation and learning transfer methods were used during the process). In the designed model, four different types of images are analyzed with four separate ResNet50 deep convolutional networks, and their extracted features are transferred to a fully connected neural network and combined with clinical and pathological features. In the model without clinical and pathological data, the maximum accuracy reached 88%, but by adding these data, the accuracy increased to 96%, which shows the significant impact of clinical and pathological data on the accuracy of diagnosis.

## Introduction

Prostate cancer (PCa) is one of the most common diseases among men. This disease accounts for approximately 15% of cancers diagnosed worldwide each year and is the second most common cancer among men^1,2^. At first, PCa is usually diagnosed by monitoring prostate-specific antigen (PSA) and Digital Rectal Examination (DER), but these methods have low specificity and sensitivity, respectively, which may cause wrong diagnosis^3,4^. Most of the time, suspicious cases are examined by Transrectal ultrasound-guided biopsy (TRUS), but this method is not accurate enough to diagnose growing cancers. In addition, this method causes 14.5% bleeding, 1% prostatitis, and 0.3% urosepsis in patients^5^. In recent studies, it has been shown that multiparametric magnetic resonance images (mpMRI) are more accurate than other methods^6,7^. In this method, images such as diffusion-weighted (DW), T2-weighted, Apparent diffusion coefficient (ADC) map, and dynamic contrast-enhanced (DCE) are used, which can act as a supplement in the diagnosis process. Normally, radiologists classify cancers according to Prostate Imaging Reporting and Data System (PIRADS-v2) from benign to malignant grade with numbers 1 to 5^8^. However, it is still difficult to diagnose the stage of the disease with simultaneous analysis of several images, and the obtained results may depend on the ability of the radiologist. Therefore, in recent years, computer-aided diagnosis (CAD) systems have been used to diagnose PCa. At the beginning of using CAD, all work was done manually, but with the advancement of artificial intelligence, these systems started using machine learning (ML). It has been shown in the research^9,10^ that the use of ML-CAD can help to diagnose the disease to some extent, but still, it cannot go through all the stages of diagnosis alone, and its input features must be extracted by humans. After the emergence of deep learning (DL), which is a subset of ML, it has been shown that this method can perform better in a large number of data. Deep learning is designed based on the function of the human brain and can extract features alone and find non-linear relationships that cannot be understood by humans. Also, Deep learning has also shown good performance in machine vision. In this research, we have used this method to diagnose the level of cancer using multiparametric images, and clinical and pathological data.

In this study, mpMRI images were used to classify patients. In the proposed method, a quadruple sequence of mpMRI images is analyzed and then clinical and pathological data are added to them to determine the level of cancer from benign to malignant with grades 1 to 5. In the initial stage, data has been collected and pre-processed. In the next step, four separate neural networks are trained for image analysis. The layers containing the features extracted from the images along with the clinical and pathological data are given as input for the final neural network (a fully connected network). In this research, the impact of using clinical and pathological data on accuracy has been investigated, which is considered the innovation of it.

The rest of the paper is organized as follow: related papers are surveyed briefly in section “Related works”. In section “Method and materials”, the proposed PCa diagnosis model constructed using deep neural network is described in detail including the description of the data set and the network architectures. The constructed diagnosis model is evaluated with respect to the main accuracy metrics such as the sensitivity, specificity f1-score and so on in section “Findings and results”. Finally, discussion of the results is provided in section “Discussion”.

## Related works

In Yueyue Wang and his colleague Manning Wang^11^ research, 9 separate CNNs have been used for 9 image models and different sequences of images have been analyzed in 5 different implementations. In the implementation, in a sequence almost similar to the current research, the accuracy of ADC, DWI (with the largest b-value), T2sag, and T2tra images was 64.9%, 67.4%, 52.6%, and 56.3%, respectively, which is an integrated model, the accuracy has also increased to 82.5%. (In this research, Bval, T2cor, Ktrans, and two other DW measurements were used). In this model, each type of image is analyzed separately in a CNN and their extracted features are merged together. In the discussion of the size of the images in this research has been shown that 64 × 64 cuts of images have better results than 32 × 32 and 128 × 128 cuts. In Yueyue Wang’s study, simple random methods such as symmetry to vertical and horizontal axes, scaling, and rotation have been used for data augmentation.

In some studies, such as Aldoj et al.^12^ and Iqbal et al.^13^, DWI with different b-values has been used. Also, some augmentation methods like transfer^13^, brightening^13^, darkening^13^, adding contamination^12,13^, symmetry to vertical and horizontal axes^12,13^, zooming^12^, rotation^13^, Gaussian smoothing^12^ have been used.

In the implementation of networks (learning part) to minimize the loss in^12^ the binary mutual entropy loss function and in^13^, mutual entropy loss function and soft Dice were used. It was shown in^12^ that AH-NET was more accurate than UNET both at the patient level and at the lesion level.

## Method and materials

In this research, all methods were carried out in accordance with relevant guidelines and regulations and all experimental protocols were approved by Trita Hospital licensing committee.

### Dataset

**Figure 1:**
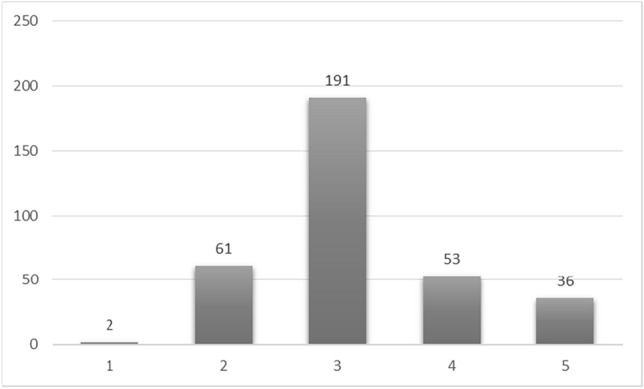
Distribution of patients according to disease progression.

In the first step, the data was collected from Trita Hospital (located in the 22nd district of Tehran, Iran). These images include T1, T2-tra, T2-sag, T2-cor, DW images (with four sizes of 50, 500, 1000, and 1500 for B-value) and ADC maps. According to previous research, T2-tra, T2-sag, DWI (with B-value = 1500), and ADC maps were selected for this study because T2 images show transition zone (TZ) area cancers better and DW images show peripheral zone (PZ) area cancers better. Also, increasing B-value has a direct relationship with increasing accuracy. For this reason, the maximum B-value has been used. (These images were produced by an 18-channel device with 10 coils receiver). Next, biopsy results were extracted from medical reports. The information obtained from the imaging reports, which included age, antigen level, dimensions, and volume of the prostate, was also collected. (Since it is difficult to diagnose prostate cancer at most levels and this disease has similar symptoms to other diseases in this area, the scientific opinions of a urology expert were used to select the factors. Prostate antigen usually shows whether the prostate is healthy or not, but when this factor is mixed with the size and dimensions of the prostate, it makes cancer distinguishable from similar diseases such as prostatitis. In addition to this, the age factor is also very influential in cancer diagnosis. For this reason, these factors are used simultaneously for diagnosis and none of them is superior to the other.) According to these reports, patients were labeled with PI-RADS-V2 scores of 1 to 5. In the end, 345 patients included all this information. (These patients visited the imaging center from October 2020 to December 2021. Informed consent was obtained from all subjects and/or their legal guardian(s)). The distribution chart of these patients is shown in Fig. 1. Also, the extracted clinical information includes the:

1. The level of PSA among patients was minimum 0.45 and maximum 230.74 and the average was 11.96 ng/ml.
2. The prostate volume among patients was minimum 12 and maximum 235 and the average was 57.21.
3. Patients’ age was minimum 39 and maximum 85 and the average was 64.05 years.

Pathological data according to Gleason scoring as two separate numbers are considered as input to introduce the progress level of the two most common cancer tissues. In Table 1, it can be seen that 122 of the patients had cancer according to the Gleason score. Also, in this table, the number of patients according to Gleason grades is presented.

**Table 1:** Distribution of patients in terms of disease level according to pathological data.

Disease level	Number of patients
3 + 3	20
3 + 4	26
3 + 5	3
4 + 3	28
4 + 4	12
4 + 5	26
5 + 4	6
5 + 5	1
The total number of patients with cancer	122

Table 2 shows the number of patients with cancer by PIRADS level. As expected, all the patients who had PIRADS 4 and 5 had clinically significant cancer, which indicates the appropriate accuracy of imaging methods in diagnosing clear cancers.

**Table 2:** The number of patients with pathologically cancer (Gleason score) by each PIRADS level.

Disease level	3 + 3	4 + 3	5 + 3	3 + 4	4 + 4	5 + 4	4 + 5	5 + 5
PI = 2	0	1	0	0	0	0	0	0
PI = 3	13	11	1	7	0	0	0	0
PI = 4	7	14	2	14	6	8	1	1
PI = 5	0	0	0	7	6	18	5	0

### Data preprocessing

**Figure 2:**
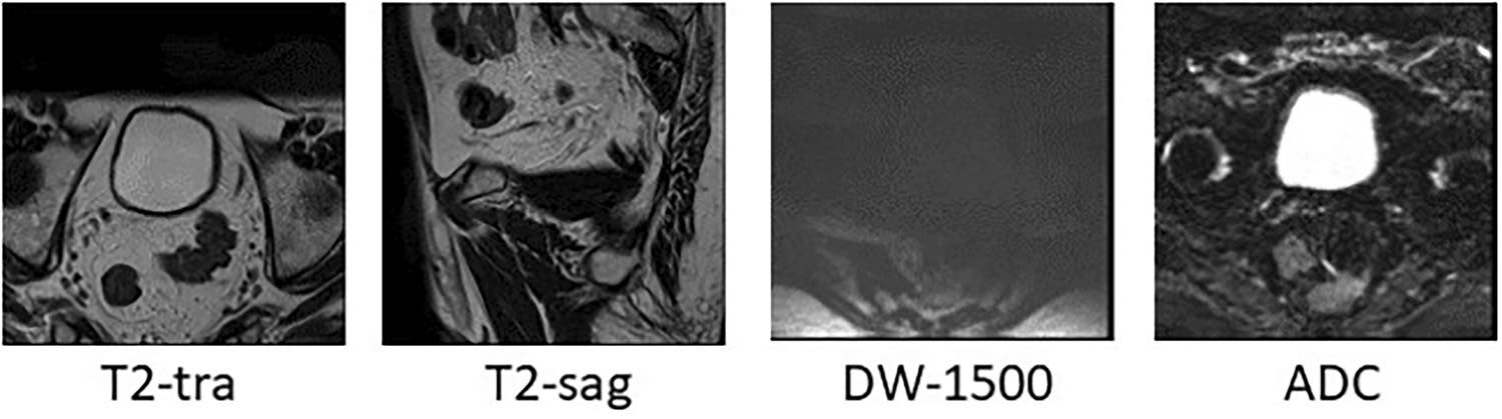
MpMRI of a level 4 patient.

In the pre-processing stage, because only two patients had label 1, this class was removed and the number of patients was reduced to 343. Then the dimensions of the images became 150 × 150. In the last step, the images were normalized. Figure 2 shows the sequence of images of a patient with stage 4 cancer. Since neural networks need a large amount of data to function properly, we had to use data augmentation techniques. Medical data is very valuable and often scarce. In addition, because we needed image data and clinical and pathological data at the same time, the number of data with these conditions was very small. The data augmentation method has been selected based on previous works (in these studies, it has been shown that the use of these techniques has increased the accuracy of the model.) flipping around vertical or horizontal axes^11,12,14^, rotation^11,12,15–17^, magnification^14^ and displacement (intensity shift)^14^ has been used in studies. In this research, with the help of the ImageDataGenerator function from the Keras library and the methods of rotation, symmetry, displacement, and magnification the data have been augmented. Flipping has been done in horizontal and vertical directions. The magnification was 0.2, and the displacement in width and length was 0.2 and 0.2, respectively. Data augmentation has been done for all data including training data and test data. In addition, because the number of data in each category (each disease level) was not the same and was much more at level 3, the model could be more inclined to learn level 3 and not perform accurately, therefore, to solve the problem, the number of images produced in the data increment is weighted. The clinical and pathological data used in this research are quantitative and qualitative variables. Quantitative variables were normalized to optimize training. Qualitative variables were prepared for analysis by the one-hot encoding method.

### Model architecture and network training

In this research, four CNN models are used simultaneously. T2-sag and T2-tra images, DWI (with B-value = 1500), and ADC maps were entered into ResNet50. After various tests, these CNNs were trained with a learning rate of 0.003, batch size of 64, in 200 epochs with the Adam optimizer function. In this research, the Relu activation function has been used in all layers of the network except the last layer which has the task of classification. In the last layer of the network, due to the four classes of the problem, the softmax function is used. Transfer learning (ImageNet) has been used in each of these networks.

**Figure 3:**
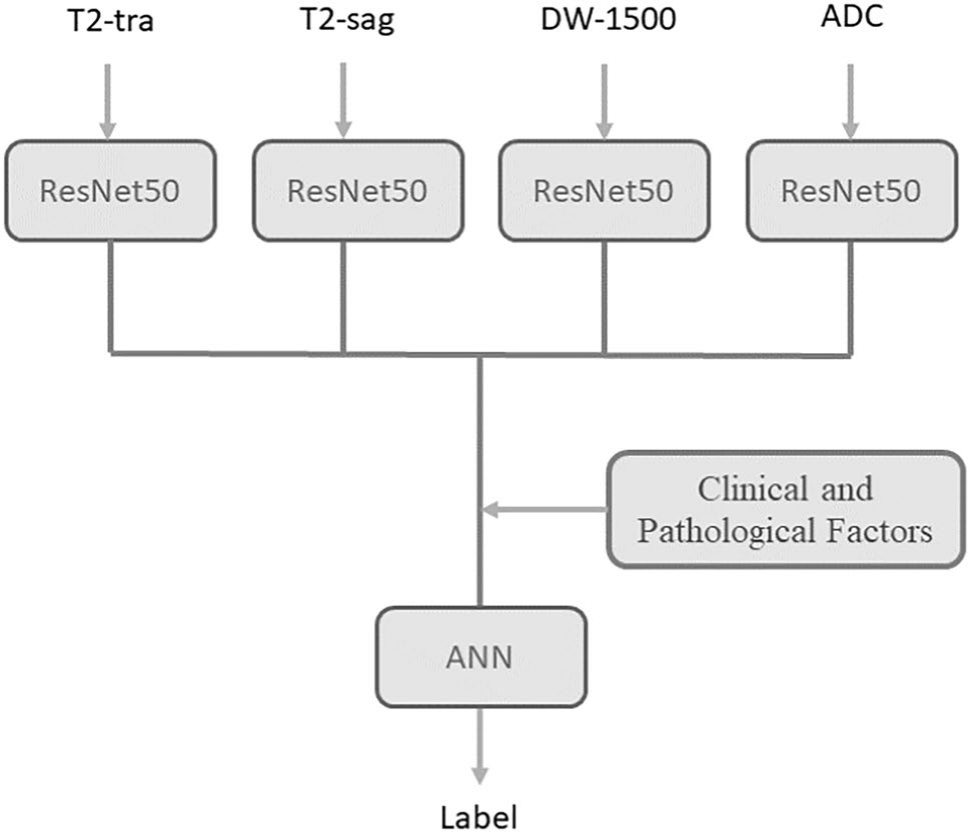
The used deep learning model.

**Figure 4:**
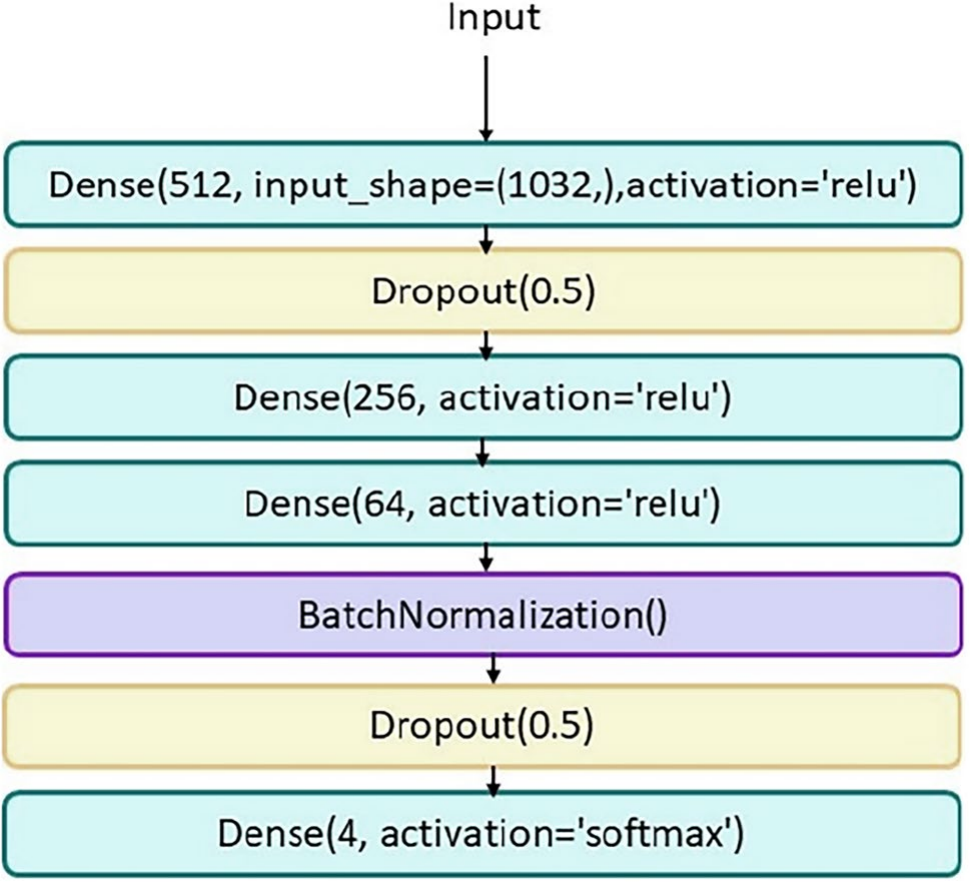
Final neural network architecture.

Then the features extracted from each type of patient image (layer before the fully connected layer) were concatenated together and added to the clinical and pathological data. As a result, a table was obtained, each record of which was a patient, in which clinical, pathological information and features extracted from T2-tra, T2sag, DW, and ADC images were included. In the next step, this table was analyzed by another neural network. This work made the information obtained from convolutional neural networks to be analyzed with clinical and pathological information. Figure 3 shows the overall final model. Figure 4 shows the architecture of the ANN network.

## Findings and results

The accuracy criteria used in this research are as follows:

- Sensitivity (Recall)
  This criterion indicates the ratio of true positive predicted observations to total actual positives.
  $${\text{Sensitivity}} = \frac{{{\text{True}}\,{\text{ positive}}}}{{{\text{True }}\,{\text{positive }} + {\text{ False}}\,{\text{ negative}}}}$$
- Specificity
  This criterion indicates the ratio of true negative predicted observations to total actual negatives.
  $${\text{Specificity }} = \frac{{{\text{True }}\,{\text{negative}}}}{{{\text{True }}\,{\text{negative }} + {\text{ False }}\,{\text{positive }}}}$$
- F1-score
  F1-score is the weighted average of precision and recall, is a criterion for evaluating the classification performance
  $${\text{F}}1{ } = \frac{{2{ * }\left( {\text{precision * recall}} \right){ }}}{{\left( {{\text{precision }} + {\text{ recall}}} \right)}}$$
- Accuracy
  This criterion indicates the ratio of true predictions to total predictions.
  $${\text{Accuracy }} = \frac{{{\text{True}}\,{\text{ positive }} + {\text{ True }}\,{\text{negative}}}}{{{\text{True }}\,{\text{positive }} + {\text{ False }}\,{\text{positive }}\,{\text{True}}\,{\text{ negative }} + {\text{ False }}\,{\text{negative}}}}$$
- Area under the curve (AUC)
  AUC represents the space under the Receiver Operating Characteristic (ROC) curve that is used to evaluate the classification model in different thresholds in terms of sensitivity and 1-specificity.

First, the results of each ResNet50 have been reviewed. For these networks, the training process was carried out during 200 iterations, and during training, early stopping was used, and at the end, the average accuracy was calculated for each one. These results are set in Table 3.

**Table 3:** Result from ResNet50 for each type of image.

Image type	ADC (%)	DW-1500 (%)	T2-sag (%)	T2-tra (%)
Accuracy	87	71	75	90

The results for the model using cross-validation (with 3 folds) without clinical and pathological data are given in Table 4 and with clinical and pathological data are given in Table 5 (We used the Chi-2 test to show the significance of the observed improvements when clinical and pathological data were included. For this purpose, we first obtained the combined frequency table and then performed the Chi-2 test with the null hypothesis of no correlation between the disease level and the clinical and pathological data, and the test hypothesis was rejected. For this reason, it has been determined that clinical and pathological data can be effective in diagnosing the level of prostate cancer).

**Table 4:** Results of cross validation for the model without clinical and pathological data.

	Accuracy
1st Fold	0.82
2nd Fold	0.88
3rd Fold	0.85
Average	0.85

**Figure 5:**
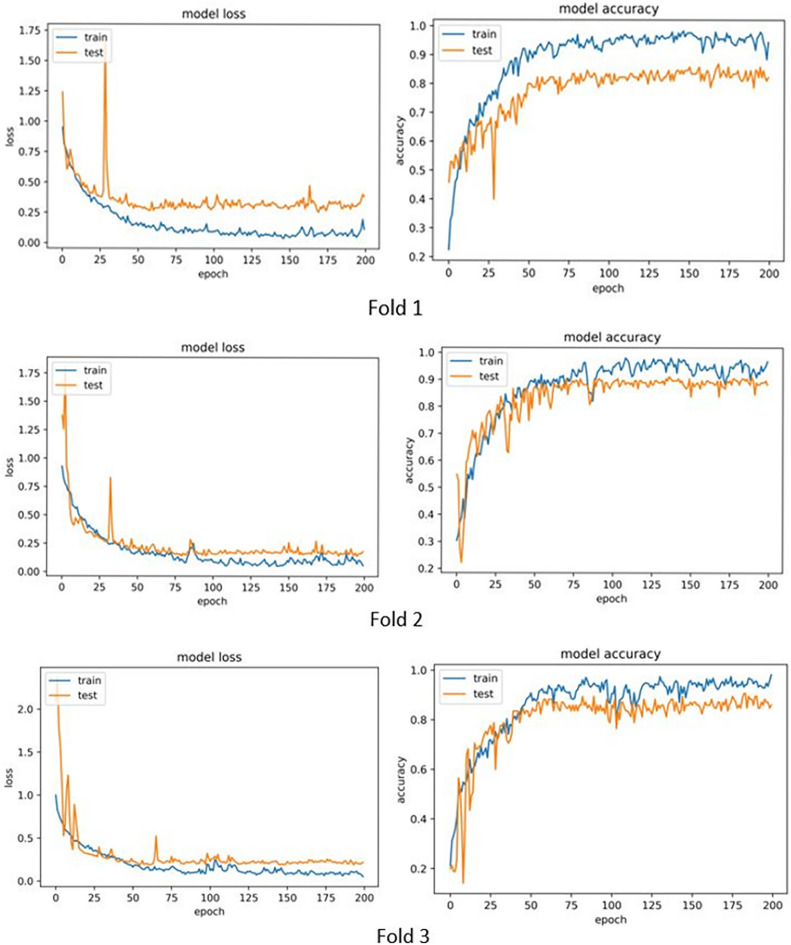
Accuracy and loss diagrams from cross validation for the model without clinical and pathological data.

The accuracy and loss diagrams of each fold are shown in Fig. 5.

As it is known, the best accuracy of 88% and average accuracy of 85% is provided by the model. In the following, similar information is provided for the model using clinical and pathological data (Table 5).

**Table 5:** Results of cross validation for the model using clinical and pathological data.

	Accuracy
1st Fold	0.94
2nd Fold	0.96
3rd Fold	0.95
Average	0.95

**Figure 6:**
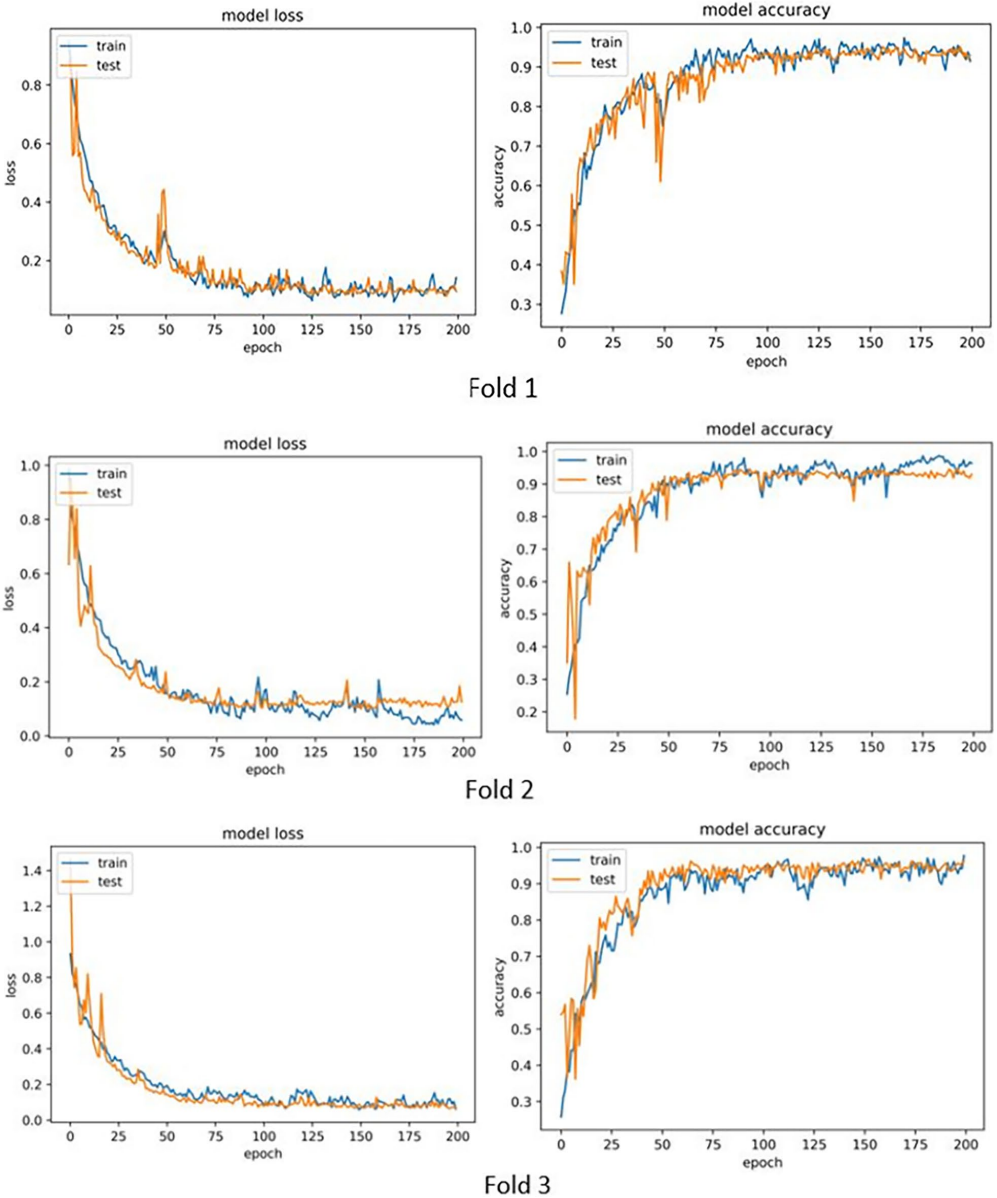
Accuracy and loss diagrams from cross validation for the model using clinical and pathological data.

The accuracy and loss diagrams of each fold are shown in Fig. 6.

As it is clear, the performance of the model has increased greatly by using clinical and pathological data. (Best accuracy 96% and average accuracy 95%).

In the following (Tables 6, 7, and 8), the accuracy and representation criteria of the model are calculated and using these two values, the F criterion can also be calculated. (This work was done by separating folds for the model along with clinical and pathological data.)

**Table 6:** Accuracy criteria for the model using clinical and pathological data in the first fold.

	Precision	Recall	f1-score
Class 2	0.97	0.84	0.90
Class 3	0.93	0.96	0.95
Class 4	0.91	0.97	0.94
Class 5	1.00	1.00	1.00
Macro avg	0.95	0.94	0.95
Weighted avg	0.94	0.94	0.94

**Table 7:** Accuracy criteria for the model using clinical and pathological data in the second fold.

	Precision	Recall	f1-score
Class 2	0.94	0.89	0.91
Class 3	0.95	0.98	0.96
Class 4	0.97	0.97	0.97
Class 5	1.00	0.95	0.97
Macro avg	0.97	0.95	0.96
Weighted avg	0.96	0.96	0.96

**Table 8:** Accuracy criteria for the model using clinical and pathological data in the third fold.

	Precision	Recall	f1-score
Class 2	0.94	0.91	0.92
Class 3	0.95	0.98	0.97
Class 4	1.00	0.86	0.93
Class 5	0.92	1.00	0.96
Macro avg	0.95	0.94	0.94
Weighted avg	0.95	0.95	0.95

In this research, the average criteria of sensitivity and specificity of the model were calculated as 94.24% and 98.62%, respectively. Prostate cancer is usually difficult to diagnose in the early stages such as 2 and 3. On the other hand, early and timely diagnosis of this disease affects the treatment process. Of course, the number of false positives should also be controlled to avoid unnecessary treatments. The sensitivity criterion shows the importance of not having a false negative, and as you can see in Table 9, this criterion is very high at level 3, which means that the number of patients who were diagnosed as healthy was acceptably low and at this level the sensitivity of the model’s performance is excellent (because at this level it is very important not to ignore the patient). On the other hand, the specificity criterion shows the importance of not having false positives, which is very high at level 2, which means that the number of healthy people who were diagnosed as sick was low. (At level 2, it is very important not to assume that a healthy person is sick.) At higher levels, both criteria were excellent, which indicates the good performance of the model at all levels.

**Table 9:** Sensitivity and specificity criteria by class.

Level of disease	Sensitivity (%)	Specificity (%)
2	8	98.43
3	97.33	97.33
4	93.33	99.06
5	98.33	99.66
Average	94.24	98.62

**Figure 7:**
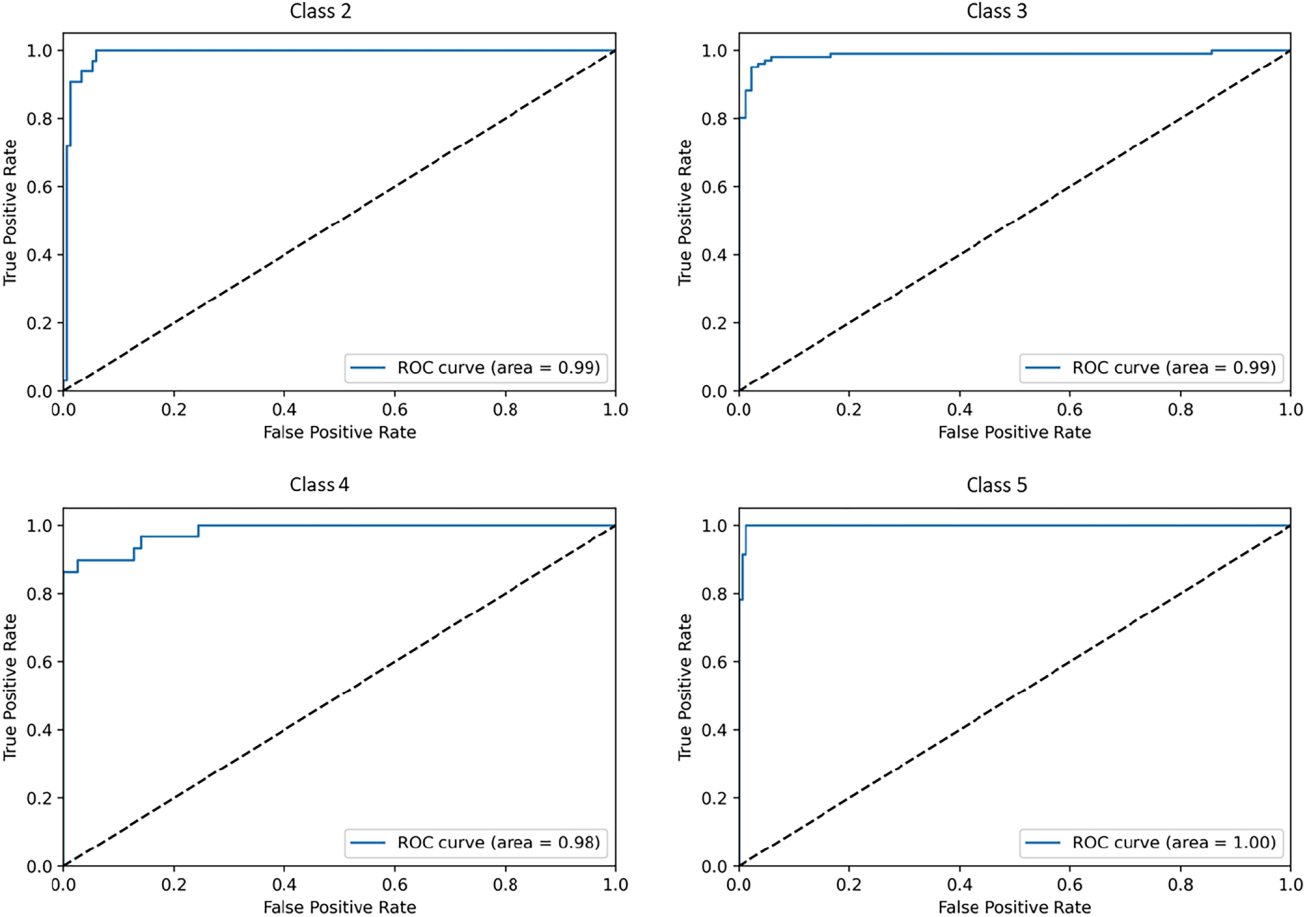
ROC diagrams for the model using clinical and pathological data by disease class.

In this part, the ROC diagram in Fig. 7 is drawn separately for each class, which shows that the performance of the model for class 5 cancers was better than the others because the area under the curve at this level is closer to one. This graph shows the ratio of true positive rate to false positive rate. For example, at level 5, it shows the ratio of the rate of patients whose disease level was correctly introduced as 5 to the rate of patients whose disease level was incorrectly considered as 5. (This work was done for the model along with clinical and pathological data.)

## Discussion

In the process of treating diseases such as prostate cancer, the time of diagnosis is always very effective. Prostate cancer in most cases has a low growth rate and shows fewer clinical symptoms in the early stages. For this reason, it is more difficult to diagnose PCa in the initial stages. In addition, the diagnosis of this disease with the help of medical images depends on the opinion of the expert, and the diversity of the observer causes the diversity of the diagnosis. Since PCa can infect other parts of the body in advanced stages, timely diagnosis is important. With the emergence of automatic diagnosis systems and the remarkable progress of machine vision, it has been shown in various studies that artificial intelligence and especially deep learning can be as efficient as an expert in diagnosing diseases similar to PCa. This research aimed to design an automatic prostate cancer diagnosis system using multi-parameter MRI and clinical and pathological data with the help of deep learning. This research was patient-oriented and presented a model that finally produces a single label for each patient's cancer level. In this research, T2tra, T2sag, DW (B-value = 1500) images, and ADC maps were used. (Since the probability of the presence of a lesion in all parts of the prostate is not the same, using different images at the same time can greatly increase the accuracy of the model. Because between 70 % and 75% of cancers occur in the peripheral region and the rest of them occur in the transition region. Also, clinical data such as PSA level in nanograms per milliliter, prostate volume, prostate dimensions, and patient's age as well as Gleason cancer score were used as clinical and pathological data. Due to the small number of data, transfer learning and data augmentation methods were used to increase accuracy. (The data used in this research were collected from Terita Hospital in Tehran). In this research, a new model was designed for the simultaneous analysis of different types of images along with clinical and pathological data. Due to the power of CNNs, this type of network was used in image analysis, and according to the results of previous studies, the ResNet50 network was selected for all four types of images. In the discussion of changing the ResNet50, the final layer of ResNet 50, which is the classification layer, is separated from it so that the features extracted from each image can be mixed with the clinical and pathological data of each patient so that the relationship between different image features and other variables can be checked in the next network. In addition, this work made the features of different images that were taken with different methods (and each one examines a different aspect of the prostate) to be analyzed together, which was an innovation of this study. In the previous works, a level of disease was reported for each image model, for example, a patient who had four images would get four results, but in our model, all the information has been investigated together and comprehensively to declare a unique number for the level of the disease (This operation was performed in two cases without clinical and pathological data and with clinical and pathological data in order to investigate the impact of non-imaging data). As expected, adding clinical and pathological data caused a big change (8%) in accuracy and showed how important it is to pay attention to other patient characteristics (The accuracy of the model was 88% without clinical and pathological data and 96% with clinical and pathological data).

Compared to previous studies, the current research has been innovative in using clinical and pathological data. Compared to Manning Wang’s research, the accuracy of CNNs used in the current research to analyze T2tra, T2sag, DW images (B-value = 1500), and ADC maps were 33.7%, 22.4%, 3.4%, and 22.1% more than their designed CNNs. The accuracy of the integration model without clinical and pathological data has also increased by 5.5%. By adding clinical and pathological data, the final accuracy difference has reached 13.5%, which shows an improvement in the cancer diagnosis process.

In addition, the average sensitivity criterion in this research for grades 2, 3, 4, and 5 was 88%, 97.33%, 93.33%, and 98.33% respectively (total average = 94.24%) which these values compared to the previous studies have been favorable and were more than many of them, which shows that the designed model is sensitive to the level of the disease.

As was shown in previous studies, the use of deep neural networks especially CNNs is useful for cancer diagnosis and they can act like an expert, for this reason, there is an alignment between the results of this research and previous studies. On the one hand, because MRI images are highly complex and every bit of information extracted from them, is very valuable, thus, it is useful to transfer information between neural network layers with the help of passing communication. It is expected that the classification accuracy will be closer to 100% as the number of patients increases. In addition, the results can be improved by designing a self-improving model. In which, it is possible to obtain visual, clinical and pathological information from users and inform them of the level of the disease at the moment. Then User data can be used to fine-tune the model.

## Limitations

One of the factors that can affect the probability and level of prostate cancer to some extent is the race and climate of the patients. The patients used in this research were from Iran and almost all of them were from Tehran. For this reason, climate change may cause a change in the obtained results (which can also be investigated in future research, but because the data available on the Internet do not have imaging, clinical, and pathological data at the same time, so far We could not provide a comparison for the effect of climate.)

Also, factors such as heredity, diet, smoking, etc. are influential in the risk of infection and the level of the disease, but unfortunately, this information was not available.

## Data Availability

The data that support the findings of this study are available from [Trita Hospital] but restrictions apply to the availability of these data, which were used under license for the current study, and so are not publicly available. Data are however available from the authors upon reasonable request and with permission of [Trita Hospital] contact: Haniye Sherafatmandjoo haniyesherafatmand@modares.ac.ir.
